# Assessment of first language adds important information to the diagnosis of language disorders in multilingual children

**DOI:** 10.1007/s40211-023-00469-w

**Published:** 2023-06-07

**Authors:** Carolin Schmid, Eva Reinisch, Claudia Klier, Brigitte Eisenwort

**Affiliations:** 1 Department of Pediatrics and Adolescent Medicine, Medical University of Vienna, Währinger Gürtel 18–20, 1090 Vienna, Austria; 2 Comprehensive Center of Pediatrics, Medical University of Vienna, Vienna, Austria; 3 Acoustics Research Institute, Austrian Academy of Sciences, Wohllebengasse 12–14, 1040 Vienna, Austria

**Keywords:** Bilingualism, Language acquisition, Typical development, Primary disease, Sociodemographic parameters, Bilingualismus, Spracherwerb, Typische Entwicklung, Primärerkrankung, Soziodemographische Parameter

## Abstract

**Objective:**

59% of Viennese day care children have a first language other than German. Lower proficiency in the second language German might be typical in multilingual settings, but might also be due to language disorder (ICD-10:F80 or comorbid). Diagnostic practise in Austria focuses on second language evaluation. This study describes a group of multilingual children with suspected language impairment at a specialized counselling hour and reflects the role of the first language in language evaluation.

**Method:**

Linguistic evaluation (typically developed, ICD-10:F80, comorbid language disorder) and sociodemographic parameters of 270 children (time period: 2013–2020) are investigated. Linguistic results are reported according to primary diseases. For children without primary disease the relation between the linguistic evaluation and sociodemographic parameters is assessed.

**Results:**

Overall, the children had 37 different first languages (74% were bilingual, 26% multilingual). The percentage of children with typical development and comorbid language development varied according to primary disease. Children without primary disease had higher chances of typical development the older they were at the examination, the earlier they produced first words, and if there was no heredity for ICD-10:F80.

**Conclusions:**

Results suggest that evaluating the children’s first language is useful since it contributes to understanding the individual language development at different linguistic levels, despite the heterogeneity of the children, and, thus, allows practitioners to recommend the best possible support.

## Abstract (de)

**Fragestellung:**

In Wien haben 59 % der Kindergartenkinder eine andere Erstsprache als Deutsch. Ein Rückstand im Zweitspracherwerb Deutsch kann im Rahmen ihrer Mehrsprachigkeit typisch sein oder aufgrund einer Sprachentwicklungsstörung (ICD-10:F80 oder komorbid) auftreten. Die Sprachevaluierung in der aktuellen österreichischen Diagnosepraxis fokussiert die L2. Vorliegende Studie beschreibt erstmalig die Gruppe mehrsprachiger Kinder, die mit Verdacht auf Sprachentwicklungsstörung in eine spezielle Sprechstunde kommen und reflektiert dabei die Rolle der Erstsprachevaluierung.

**Methodik:**

Linguistische Evaluierungsergebnisse (typisch entwickelt, ICD-10:F80, komorbide Sprachentwicklungsstörung) und soziodemographische Parameter von 270 Kindern (Untersuchungszeitraum: 2013–2020) wurden deskriptiv ausgewertet. Die linguistischen Ergebnisse wurden nach Grunderkrankungen aufgeschlüsselt. Bei Kindern ohne Grunderkrankung wurde der Zusammenhang zwischen linguistischem Ergebnis und soziodemographischen Parametern berechnet.

**Ergebnisse:**

Insgesamt 37 verschiedene Erstsprachen wurden von den Kindern gesprochen (74 % bilingual, 26 % mehrsprachig). Bei Kindern mit Grunderkrankung variierte der Anteil der Kinder mit typischer Entwicklung und komorbider Sprachentwicklungsstörung je nach Grunderkrankung. Kinder ohne Grunderkrankung hatten größere Chancen auf typische Entwicklung, wenn sie bei der Untersuchung älter waren, früher erste Worte sprachen, und keine familiäre Häufung von ICD-10:F80 vorhanden war.

**Schlussfolgerungen:**

Die Studienergebnisse weisen darauf hin, dass eine Erstsprachevaluierung sinnvoll ist, um trotz der Heterogenität der vorgestellten Kinder valide Daten zum individuellen Sprachstand auf verschiedenen linguistischen Ebenen zu gewinnen und eine bestmögliche Förderung zu empfehlen.

## Introduction

Vienna is a melting pot of different languages and cultures. Approximately 60% of all Viennese children grow up with a first language (L1) other than German [1]. In multilingual settings, there are many factors which influence language proficiency like cross-language influence, or quantity and quality of language input in L1 and L2 (second language) [2]. Thus, even healthy children vary in their speed of L2 acquisition, that is, in their L2 proficiency at a given time. A lower L2 proficiency compared to monolingual children often leads professionals to consider a language impairment. Superficially, L2 speech patterns in early stages of acquisition may resemble patterns observed in impaired monolingual language development [3]. Lower L2 proficiency in multilingual children may hence be a normal phenomenon in the framework of L2 acquisition. However, for some children, it might indeed be traced back to a language disorder (LD [4]).^1^ Because of the strong variation between children (e.g., L1, number of languages regularly spoken, [socio-]psychological factors) and the resultant complexity in the evaluation of the children’s language development, a comprehensive analysis including a language assessment in the children’s L1 can add important information (see also [5]).

An LD might occur in otherwise healthy children, or in children with primary disease. Depending on the type of disease, a comorbid language disorder (CLD) can be expected, for example, in the case of autism spectrum disorder (ASD, ICD-10:F84, [6]). In other cases, the primary disease is not directly associated with an LD, for example in some metabolic diseases. However, children with primary diseases must often manage a challenging daily life, so that the primary disease can be paramount. In case their primary disease is accompanied by a CLD, the children additionally have to compensate for their linguistic deficits in communication.^2^

An evaluation of a suspected LD in multilingual children is important. A falsely ascribed LD might have far-reaching consequences for the child’s self-esteem and academic performance, while children with an actual LD need speech therapy. Unfortunately, in our clinical experience in Austria, clinical routines and psychological practice often only involve the assessment of the L2 German according to monolingual norms. However, the mere focus on the L2, and, specifically, on monolinguals as reference can lead to an overdiagnosis of LD (see also, e.g., [7]). Therefore, many studies on multilingual children with a focus on differentiating between typical development (TD) and LD suggest the combination of L2 measures (e.g., nonword or sentence repetition tests) with a parent questionnaire about the L1 development [8]. This procedure is readily applied by professionals because they do not need any knowledge of the L1. For children without any observed sensory, motor, or neurological impairment who are able and willing to cooperate in the test procedure, this combination of L2 assessment and parent questionnaires might be sufficient to establish whether their language development is typical or not (ICD-10:F80 [20]). Generally, the validity of parent questionnaires is debatable. Even in cases of L1 development, parent questionnaires often lead to false assessments [9, 10]. In an L2 setting, many families are in a psychosocially burdening situation because of their low social status in the majority society, due to socioeconomic problems or sometimes to traumatic experiences related to their flight. In this situation, parents’ information about their children’s language development might be even less reliable. Many studies show that in order to speak of an LD, the disorder must occur in all languages and in all communication situations [3]. Therefore, testing in all languages must be seen as the gold standard. It allows for the classification of the language development of multilingual children in TD and LD.

In Vienna, multilingual children with a suspected LD can be examined in a specialized counselling hour. During this counselling hour, a linguistic evaluation of the children’s L1 and L2 development is conducted and interpreted based on information about the children’s individual multilingual situation. These results are combined with results of a psychological evaluation and integrated into a pediatric perspective.

The goal of the present study is to present an overview of the children who visited this counselling hour between 2013 and 2020. By looking at their language, medical, sociodemographic, and psychological parameters, we aim to provide a solid basis for diagnosis and thereby to recommend the best possible support.

### Specific aims of the study

The first aim is to describe linguistic evaluation results and sociodemographic parameters of the group of children sent to the counselling hour. The second aim is to assess the role of potential primary diseases in the linguistic evaluation of said children. This represents an important contribution because, to the best of our knowledge, no other data is yet available on the relationship between a potential primary disease and the linguistic evaluation result in multilingual children with a suspected LD. For children with a primary disease, the description focuses on the categorization of primary diseases and their relation to the linguistic evaluation. For children without primary disease the description focuses on sociodemographic parameters possibly predicting the linguistic result.

## Methods

### Database and data collection

In 2012, a counselling hour for multilingual children with suspected language disorder was established. In order to determine whether there is evidence for an LD in all of the children’s languages, the procedure of the examination typically comprises a case history and a description of their L1 and L2 proficiency. To the best of our knowledge, this counselling hour is the only institution in Austria offering a systematic L1 assessment in addition to an assessment in German. The L1 assessment is conducted by linguists together with native speakers of the respective language who are trained in medical communication. This is facilitated by medical students with L1s other than German. Results of the L1 and L2 assessment are integrated into the evaluation of the children’s development as given by the psychological report.

All children are requested to attend two appointments with their parents or caregivers.

During the first appointment, a parent interview is conducted (based on, e.g., [11]) as well as a description of the child’s L1 development, each with the aid of a native speaker. More detail regarding the parent interview is shown in Table 1, while the ideal systematic assessment of the children’s receptive and expressive L1 development is shown in Table 2. Normed tests and screening procedures such as [11–14] are used.

**Table 1:** The questions included in the case history are based on multiple questionnaires (e.g., [11])

Obligatory questions	Further questions
Course of pregnancy and birth	Chosen and adapted on an individual basis (as linguistic and social situations may vary between children)
Previous medical reports (about possibly relevant primary diseases)	
Possibility of heredity of LD	
Age at first word production	
Synchronous language use	
Diachronous language use	

*LD* language disorder

Heredity is defined as family aggregation of language impairments (according to ICD-10:F80) as reported by the parents [22, p. 33]. First word production is defined as the production of the first two words besides “mummy” and “daddy”, e.g., “car” or “doll”

**Table 2:** Language tests and screenings ideally performed in the framework of the counselling hour, according to children’s age and split by first language (L1) and second language (L2), and by language reception and expression. In addition, language independent nonword repetition tests are performed in order to test phonological awareness and to have a further index of typical/atypical language development. In case of atypical articulation, articulation is further tested systematically, if possible, in L2 German, and in a spontaneous speech sample in the L1. The age-dependent milestones are analyzed at the phonetic–phonological level (phonological discrimination, phonological processes), the morphosyntactical level (number of words within a sentence, word order, complexity of sentences and subclauses, case, gender, and plural markers, subject–verb congruency), and the semantic–lexical level (amount of different words, word classes), with reference to descriptions for German (see also summary in [14]) and to further language-specific and universal developmental patterns (e.g. [31]). See bibliography for full names of tests and screening procedures

Age (in years)	L1		L2			
	Reception	Expression	Reception		Expression	
Always	PPVT‑4 screening [12]		PPVT‑4 [12]			
	Spontaneous speech conversation (with/without use of picture books/stories/toys), with respect to the age-dependent milestones for the specific linguistic levels in receptive and expressive language development		Spontaneous speech conversation (with/without use of picture books/stories/toys), with respect to the age-dependent milestones for the specific linguistic levels in receptive and expressive language development			
3	Russian language proficiency test for multilingual children [11]	Russian language proficiency test for multilingual children [11]	Lise-DaZ [15]			
4						
5	Havas 5, for Italian, Polish, Portuguese, Russian, Spanish, Turkish [13]					
6						
7						
8	–	–	ADST [17]	HSET [16]	ADST [17]	HSET [16]
9	–	–				
10	–	–		–		–
11	–	–		–		–
12	–	–		–		–
13	–	–		–		–
14	–	–		–		–
15	–	–		–		–
16	–	–		–		–
17	–	–	–		–	
18	–	–	–		–	

During the second appointment, the focus is on the L2 German development (Table 2 shows test and screening procedures [12, 14–17]). The actual individually applied procedure always depends on the age of the child, as well as on L1/L2 experience of the child (in terms of quantity and quality of L1/L2 use), screening procedures available in L1, and on the child’s ability to participate in the respective tests.

**Fig. 1:**
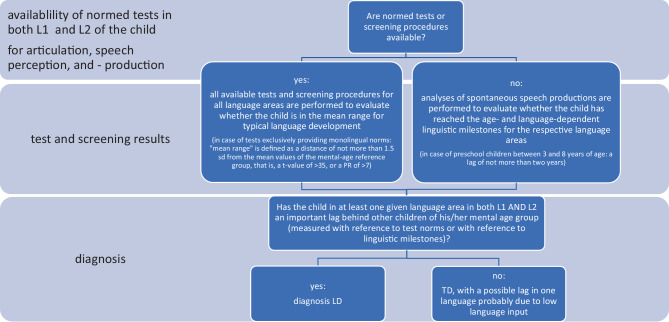
Description of the basic prerequisites for a diagnosis of language disorder (LD). *L1* first language, *L2* second language, *sd* standard deviation, *PR* percentage range, *TD* typical development

At the end of the second appointment, counselling for parents about the results of the linguistic evaluation takes place, that is, about the results of the language assessments interpreted within the framework of the children’s individual multilingual situation, against the background of their psychological report. See Fig. 1 for a description of the basic prerequisites for a diagnosis of LD.

For further information about the examination procedures used in this counselling hour, see [18, 19].

### Data preparation

The data of children who were examined at the counselling hour between 2013 and 2020 were manually reviewed and entered into an anonymized database, including the sociodemographic parameters from the case history (Table 3).

**Table 3:** Sociodemographic parameters from the case history that were entered into the anonymized database for further analyses

Variable	Type	Values
L1	Nominal	All first languages of the children; language varieties were subsumed, e.g., Tunisian and Syrian Arabic to “Arabic”
Number of regularly used languages	Nominal	Two, more than two
Gender	Binary	Female, male
Heredity of ICD-10:F80	Nominal	Yes, no, unknown
Age at time of first examination 1	Metric	Age (in months)
Age at time of first examination 2	Metric	< 42 months, i.e., about the start of day care; 42–90 months, i.e., around school entry; > 90 months
Age at time of first word production 1	Metric	Age (in months)
Age at time of first word production 2	Metric	< 13 months, 13–24 months, > 24 months
Existence of a primary disease	Binary	Yes, no
Specification of the primary disease	Nominal	Intellectual disability, hearing impairment, neuropediatric disease, somatic disease, psychiatric disorder (except ASD), psychiatric disorder (diagnosed/suspected ASD)

*ASD* autism spectrum disorder (ICD-10:F84), *suspected ASD* ASD is suspected by a clinical psychologist, but the autism-specific assessment is not concluded yet (as considerable time may elapse from the first suspicion until the result of an ASD-specific assessment), *L1* first language

Based on the linguistic evaluation and further medical and psychological reports, children were divided into three groups of linguistic results: children with TD, children with ICD-10:F80, and children with CLD.^3^ Children with CLD belong to one of the categories of primary diseases outlined in Table 4.

**Table 4:** Categorization of primary disease with inclusion and exclusion criteria

Category		Label	Inclusion criteria	Exclusion criteria
1		Intellectual disability	Children scoring below average range in nonverbal cognitive ability, regardless of somatic diseases	Diagnosed or suspected ASD
2		Hearing impairment	Children with a mild, moderate, or severe hearing impairment, regardless of somatic diseases	Nonverbal cognitive ability below average range
3		Neuropediatric diseases	Children with a neuropediatric disease like epilepsy or neurofibromatosis	Nonverbal cognitive ability below average range
4		Somatic diseases	Mainly children with metabolic diseases	Nonverbal cognitive ability below average range
5	5a	Psychiatric disorders, according to ICD-10, chapter V (F)	–	Nonverbal cognitive ability below average range, diagnosed or suspected ASD
	5b		Children with diagnosed or suspected ASD, regardless of their nonverbal cognitive ability	–

*ASD* autism spectrum disorder (ICD-10:F84), *suspected ASD* children, for whom ASD is suspected by a clinical psychologist, but the autism-specific assessment is not concluded yet (as considerable time may elapse from the first suspicion until the result of an ASD-specific assessment)

### Data analyses

For statistical analyses, the software R was used ([21], version 4.0.0). First, an overall description of the group of children presented at the counselling hour is given. Second, the subsets of children with and without primary disease are described. The frequencies of the linguistic results and the values of sociodemographic parameters were calculated for all groups. To test for a correlation between the parameters gender and L1, linguistic result and L1, and linguistic result and the groups of children with and without primary disease, chi square (χ^2^) tests of independence were calculated. For children without primary disease, additional bivariate logistic regression models were used to investigate the predictability of the linguistic result.

## Results

### Overall description of the group of multilingual children

**Fig. 2:**
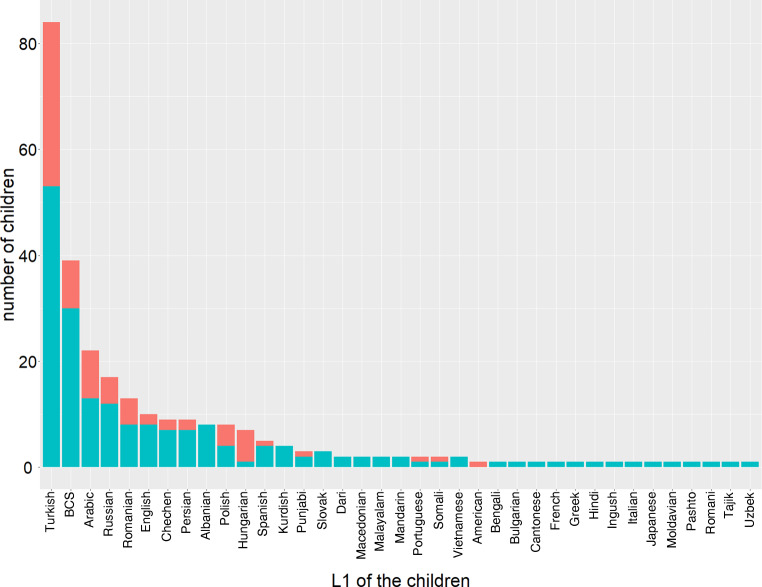
Frequency (in number of children) of different first language (L1) of the children examined at the counselling hour; *orange* frequency of female children, *turquoise* frequency of male children. *BCS* Bosnian/Croatian/Serbian

In total, 270 children were examined at the counselling hour, and 37 different L1s were assessed (Fig. 2). The most frequent L1s were (in order of descending frequency): Turkish, Bosnian/Croatian/Serbian, Arabic, Russian, and Romanian. A total of 168 children had one of these five languages as their L1. The most infrequently represented 15 languages were each spoken by only one child. 74% of the children used only one language in addition to their L2 German, 26% multilingual.

The gender gap (70% male vs. 30% female children) does not differ between the five most frequent languages (χ^2^(5) = 7.19, *p* = 0.207).

The age of the children at their first visit ranged from 1;6 to 19;0 years. An analysis of the three age groups mentioned above showed that 16% of all children were examined at an age younger than 3;6, 61% of children were examined between 3;6 and 7;6, and 23% of children were examined after the age of 7;6.

An analysis of the onset of speech production shows that 46% of all children produced first words at around 12 months of age, 33% of the children started to produce words within their second year of life, and 21% of the children produced their first words after their second birthday.

**Fig. 3:**
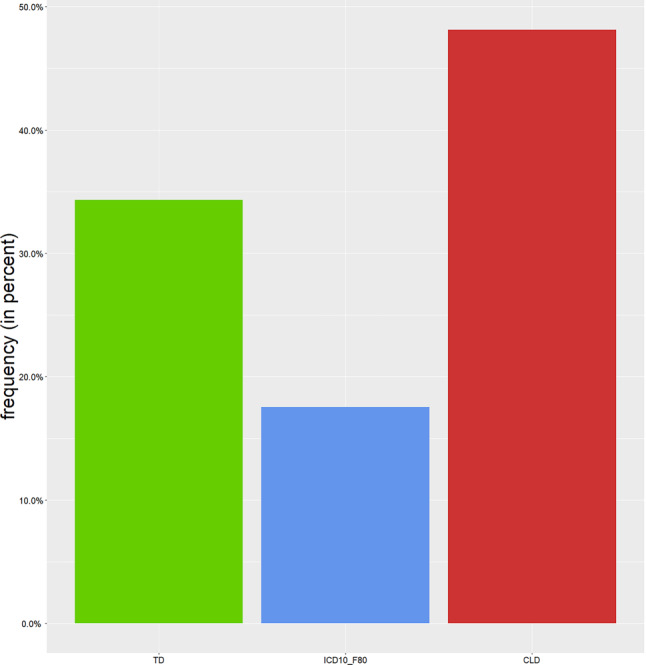
Frequency (in percent) of linguistic evaluation results of the whole group of children examined at the counselling hour; *green* typically developed (TD), *blue* language disorder in the framework of ICD-10 : F80 (ICD10_F80), *red* comorbid language disorder (CLD)

Having provided some general information about the children examined at the counselling hour, the next section is concerned with the question of LD. Figure 3 shows how many children received the result TD, ICD-10:F80, and CLD, respectively.

When the composition of these three groups of children is analyzed with respect to the variable L1 (restricting the analysis to the five most frequent L1s listed above), no significant difference in the linguistic evaluation depending on L1 was found (χ^2^(10) = 6.09, *p* = 0.808).

### Children with primary disease

Children with a previously determined primary disease represented 53% of all children. Of these children 90% were linguistically evaluated with a CLD, and 10% received the result TD. Table 5 shows these results split by the different categories of primary diseases. In all groups, the number of children with CLD was higher than the number of children with TD. The highest number of children with CLD was found for ASD (100%), and for intellectual disability (97%). The lowest number of children with CLD (61%) was found for somatic diseases.

**Table 5:** Categorization of children with primary disease according to the group of disease. The absolute number of children within the respective groups, as well as the percentage of children with CLD and TD for each group is given

Primary disease	CLD (in %)	TD (in %)	Number of children (female/male)	Age (mean [SD]) in months
1: intellectual disability	97	3	37 (14/23)	71 [29]
2: hearing impairment	83	17	6 (2/4)	84 [40]
3: neuropediatric disease	80	20	10 (3/7)	86 [49]
4: somatic disease	61	39	18 (6/12)	70 [43]
5a: psychiatric disorder (except ASD)	79	21	19 (5/14)	77 [45]
5b: psychiatric disorder: diagnosed/suspected ASD	100	0	54 (12/42)	53 [17]

*CLD* comorbid language disorder, *TD* typical development, *ASD* autism spectrum disorder (ICD-10:F84), *suspected ASD* children, for whom ASD is suspected by a clinical psychologist, but the autism-specific assessment is not concluded yet (as considerable time may elapse from the first suspicion until the result of an ASD-specific assessment), *SD* standard deviation

### Children without primary disease

Children without primary disease represented 47% of all children. Of these children, 62% were eventually evaluated as TD and 38% received the result ICD-10:F80. These frequencies of linguistic results differed significantly from the frequencies in children with a primary disease (χ^2^(2) = 217.5, *p* < 0.0001).

For 63% of the children without primary disease, no heredity for ICD-10:F80 was indicated, whereas for 28%, a heredity was known. In the remaining 9% of the families, heredity could not be determined.

#### Parameters influencing the linguistic result of children without primary disease

In this section, we test whether and to what extent the sociodemographic parameters *L1, number of languages regularly used, gender, heredity, age at first examination* (in months), and *age at first word production* (in months) predict the result of the linguistic evaluation (TD, ICD-10:F80) in children without primary disease. To this aim, logistic regression models with the dependent variable *linguistic result* were calculated using the function generalized linear models (glm).

In order to analyze the influence of different L1s on the dependent variable *linguistic result*, a model was built on a reduced dataset which only included the four most commonly used L1s in the present study (Turkish, Bosnian/Croatian/Serbian, Arabic, Russian). This decision was made because all other L1s were each spoken by fewer than 5 children without primary disease. The effect of L1 was evaluated by building a model with L1 and all other parameters as independent variables and comparing the fit of this model to one without the factor L1. Goodness of fit was evaluated using the Akaike information criterion (AIC), which is an estimate of model quality and combines the goodness of the model fit and model complexity to prevent overfitting. Results showed that the *L1* did not contribute to predicting the linguistic result (model including L1: AIC = 91.87 vs. model without L1: AIC = 87.62).

Next, the influence of the remaining parameters on *linguistic result* was analyzed. Since L1 appeared not to predict the outcome, a new model was built on the complete dataset including all L1s and excluding the factor L1. In order to test the contribution of each independent variable on the predictive power of the model, the R function drop1 was used. This function eliminates variables one-by-one and compares the models’ fit using the AIC. Removing the variable *number of languages regularly used* resulted in a better AIC (model including factor: AIC = 132.05, model without factor: AIC = 130.09). Removing additional variables did not result in further improvements according to the AIC selection criterion. Results of the best fitting model are shown in Table 6. The predictor variables with significant coefficients were *heredity, age at first examination*, and *age at first word production*. Children without heredity had 3.5 times higher odds of receiving the result TD than children with a known heredity, all other factors being equal. The older the children were at their first examination, the more likely they were to receive the linguistic result TD. With each one month increase in age, the odds of receiving the result TD increased by 1.0186. For the variable *age at first word production*, in contrast, increasing age was related to lower chances for TD. Here, with each one month increase in age, the odds for the result TD decreased by 0.917.

**Table 6:** Results returned by the bivariate logistic regression model that best fit the linguistic results obtained by the multilingual children without primary disease examined at the counselling hour. Linguistic result is the dependent variable (TD vs ICD-10:F80), and gender (female vs. male), heredity (yes, no, unknown), age at first examination (continuous variable, in months), and age at first word production (continuous variable, in months) are independent variables

	b	SE	Z value	*p*-value	OR	95% CI (LL;UL)
(Intercept)	0.32	0.89	0.36	0.7166	1.383	0.2433; 8.3717
Gender (m)	−0.82	0.52	−1.59	0.113	0.4412	0.1527; 1.179
Heredity_no	1.26	0.51	2.48	0.0131	3.5079	1.3205; 9.7234
Heredity_unknown	2.53	1.18	2.15	0.0318	12.5078	1.7156; 263.479
Age_examination	0.02	0.01	2.09	0.0371	1.0186	1.0019; 1.0374
First_words	−0.09	0.03	−2.95	0.0032	0.9172	0.8623; 0.9683

*TD* typical development, *b* estimate, *SE* standard error, *OR* odds ratio, *CI* confidence interval, *LL* Lower Limit, *UL* Upper Limit

## Discussion

The aim of the present study was to describe the group of multilingual children who are suspected of having an LD and are therefore sent to a specialized counselling hour. In addition to an overall description of the whole group of multilingual children who were examined, two groups of children were differentiated: one without primary disease (47% of all children) and one with primary disease (53% of all children).

As described above, a primary disease can often be directly or indirectly associated with CLD. In fact, our results show that a suspected LD was confirmed more often in children with primary disease (90% of these children) than in children without primary disease (38% of these children). This reflects the differences of the prevalence of LD described in the literature. For ICD-10:F80, a prevalence between 5–8% is indicated [22], whereas for children with primary disease, a prevalence of CLD of up to 20% is suggested [23]. Even though the suspected LD can be rejected for only 10% of children with primary disease, they have a wide spectrum of diseases, with some of them more strongly associated with LD than others. Since an LD is expected in many young children with ASD [6], the L1 evaluation only provides information about the pattern of the disorder [19]. Many further diseases are characterized by a high incidence of CLD (e.g., epilepsy [24]), so that the probability of confirming a CLD in the children’s L1 is high. In contrast, many somatic diseases like juvenile diabetes are not directly associated with LD. However, a general association of psychological disorders with juvenile diabetes is observed (e.g., depression, anxiety disorders, [25]), which might enhance the probability of CLD. Nevertheless, for 39% of children with somatic diseases in our study, the suspected LD could be rejected, as well as for about 20% of children with neuropediatric diseases, psychiatric diseases (except ASD and cognitive disorders), or hearing impairments. Thus, it is important to critically investigate the suspicion of an LD, despite a generally strong association between primary diseases and LD.

Significant predictors for the linguistic result for children without primary disease were *heredity of ICD-10:F80, age at first word production*, and *age at first examination*. Children without heredity have better chances of TD than children with known heredity, which is in line with the literature (e.g., [26]). Children who produce their first words later have a higher risk of having ICD-10:F80. This result was also expected, as typically developed children mostly produce first words around 12 months, whereas children with LD often produce first words later (e.g., [27]), despite large individual variation. Results further showed that the older the children were at their first examination, the more likely they were to receive the result TD. Older children are probably more often examined as a precaution, often ordered by the school. In contrast, if younger children are sent for language assessment, there might be a higher suspicion of an LD, often noticed by the parents themselves.

The overall distribution of linguistic results (TD, ICD-10:F80, CLD) was independent of L1 and gender. This reflects that “language development in children is remarkable for its regularity across individuals and different languages, even when the languages are quite diverse” [28]. Children’s L1s were distinctly heterogeneous. The most frequent L1s were Turkish, Bosnian/Croatian/Serbian, Arabic, Russian, and Romanian, which roughly reflects the overall distribution of non-German L1s in Vienna [1]. Typologically, these languages differ at various linguistic levels, leading to different cross-language influences on L2 German. This variety of L1–L2 patterns is further highlighted by the many L1s that were represented with sometimes as few as one child per language. In addition, 26% of the children examined in this study are regularly exposed to more than two languages. Although this increases the possibilities of cross-language interference (L1–L2–L3) and likely reduces the amount of input in each of the languages, the number of languages spoken did not predict the number of children referred to the counselling hour (nor did it predict the linguistic results, looking at the children without primary disease).

In Austria the majority language has a high value in the educational system. Because L2 German assessments are part of the obligatory school entrance tests [29], a large proportion of the children present at the counselling hour in temporal context with school entry. Our results show that these children are more often unwarrantedly suspected of having LD than younger children. A comprehensive language evaluation including an L1 evaluation might correct this misjudgement without further negative consequences.

To our opinion the common practice to focus on an L2 assessment cannot completely meet the children’s needs. An LD must be verified in each of the children’s languages. Many children in our study received the result TD on the basis of their L1 and their specific multilingual setting. Children with a L1 other than German represent 59% of all children between 3 and 5 years in Viennese day care [1]. Therefore, all languages should be recognized and valued (see also [30]). A mere assessment of the L2 is not sufficient, because many multilingual children are actually behind monolingual children in their L2 German (see, e.g., [22, p. 33]), which has also been confirmed by the evaluations in the counselling hour. However, this does not justify the diagnosis of an LD. Moreover, none of the investigated parameters, that is, neither the respective primary disease nor the various sociodemographic parameters, can explain the outcome of the linguistic result (TD, ICD-10:F80, CLD) by itself. These parameters increase the chances of a given result, but the linguistic evaluation, including an L1 assessment, cannot be replaced by this information. In addition to the classification of the language disorder, the linguistic assessment allows the description of patterns of the disorder and thereby the development of strategies for therapy. Whenever possible, native speakers should be integrated in order to analyze the child’s language together with linguists and the child’s parents. We assume that the inclusion of native speakers would indeed be possible at many locations. A high percentage of (university) students acquired other first languages than German and might therefore contribute to the L1 assessment process, as well as a high percent of day care staff. Moreover, children and their families might benefit from the integration of native speakers as a cultural broker.

## Footnotes

1 We use the term “language disorder” (LD) as a generic term for children with persistent language problems independently of whether they have a primary disease or not. This term has also been accepted by panel members of the Delphi process [4, p. 674].

2 In the following, we use the term “primary disease” and the term “comorbid language development disorder” instead of “co-existing condition” and “language disorder associated with x” (see [4, p. 677]).

3 Particularly in the first years of the counselling hour, psychological reports were sometimes missing. However, when a suspected LD was confirmed, the result ICD-10:F80 was only given in case of age-adequate global development without indication of comorbidities according to ICD-10, V.
